# Supplementary material for: Engaging community pharmacists to eliminate inadvertent doping in sports: A study of their knowledge on doping
Source: PLoS One. 2022 Jun 10;17(6):e0268878. doi: 10.1371/journal.pone.0268878 (PMC9187095; doi:10.1371/journal.pone.0268878)
Supplement: Attachment — Submitted filename: review-PLOS_ Pharmacists.pdf [file pone.0268878.s004.pdf]

The article is interesting and relevant. However, it lacks of rational and the results are very descriptive while they could be linked to hypothesizes. Another weakness is the unbalanced sampling regarding for example age, gender or academic levels. Indeed it's unclear if this sample is representative of the Malaysian pharmacists.

The introduction is too short and the research question is the question is insufficiently problematized: It is not clear why pharmacists are in the first line regarding doping ? The examples provided in the beginning of the MS did not indicate that the pharmacists were involved in inadvertent doping. Another key point is the cultural definition of the pharmacists wok in Malaysian that is not defined. Indeed, it is expected the potential role of the pharmacists could change between countries: from physician collaboration, patient counselling to commercial benefits… Please explain how pharmacists work in Malaysian: are there able to sell drugs without physicians order? Is there durgs that are free to sell even they are prohibited in the WADA list? Is there a commercial activity linked to the price of drugs that offers potential differential benefits between pharmacists' office? There is no review of research and most of the references are only proposed in the discussion. In addition, the question of assessment of the knowledge is not considered: what types of knowledge? How they could be assessed ? In the result section, the sample has been split and comparisons using different variables such academic level, year of practice and so on. The reason why these comparisons have been done and are relevant should be explain in the introduction. Are there Hypothesizes for that regarding previous research? In addition in the introduction, it will be also interesting to indicate their potential role for doping violation rule regarding trafficking of prohibited substances to athletes and unintentional intake of a prohibited substances.
To resume, this article did not provide a sufficient report of what you know now and what we need to know that justify this study.

The results are however interesting and the discussion and conclusion clear. Please see if you can also adjust the sample or justify its representativness.
