# Supplementary material for: Engaging community pharmacists to eliminate inadvertent doping in sports: A study of their knowledge on doping
Source: PLoS One. 2022 Jun 10;17(6):e0268878. doi: 10.1371/journal.pone.0268878 (PMC9187095; doi:10.1371/journal.pone.0268878)
Supplement: Attachment — Submitted filename: Cover Letter_Response for Resubmission.docx [file pone.0268878.s005.docx]

**Date: 15^th^ October 2021**

**Professor Dr Muhammad Shahzad Aslam**

**Academic Editor**

**PLOS ONE**

Dear Editor,

**Manuscript: Engaging Community Pharmacists to Eliminate Inadvertent Doping in Sports: A Study on their Knowledge on Doping**

We thank the reviewers for their constructive comments. We have revised and proofread the manuscript accordingly. Important changes are annotated with ‘comments’ in the manuscript. Additions are highlighted in yellow; other types of edits are marked with ‘Track Changes’.

**Reviewer #1:**

1. **The article is interesting and relevant. However, it lacks of rational and the results are very descriptive while they could be linked to hypothesizes.**

***Response to reviewer:*** The main aim of the study is to gauge the current level of knowledge of doping among community pharmacists. This will in turn inform the design of future studies involving specific interventions among pharmacists based on proven hypotheses. Thus, the descriptive nature of the study is inevitable, as there is a lack of baseline data to form a hypothesis.

2. **In the result section, the sample has been split and comparisons using different variables such academic level, year of practice and so on. The reason why these comparisons have been done and are relevant should be explain in the introduction. Are there Hypothesizes for that regarding previous research?**

***Response to reviewer:*** We have expanded the introduction and added studies describing the effects of prior training on doping-related knowledge levels: “Previous studies reported that specific training, such as university courses and workshops, significantly influenced respondents’ levels of doping-related knowledge [1,2,3].”

The findings of these studies suggest that academic qualifications or years of experience may affect one’s level of doping-related knowledge. We have also included the findings of these studies in the discussion.

3. **Another weakness is the unbalanced sampling regarding for example age, gender or academic levels. Indeed it’s unclear if this sample is representative of the Malaysian pharmacists.**

***Response to reviewer:*** We have expanded the description of the methods to include the study design and the sampling procedure. Also, we have added descriptions of the findings of other studies showing that the demographic characteristics reported by those studies are similar to ours.

“In terms of the demographic characteristics, we found that the majority of the respondents were young Chinese women with professional degrees in pharmacy. This is in line with the characteristics of the participants enrolled in other studies involving community pharmacists in Malaysia [4,5,6].”

4. **The introduction is too short and the research question is insufficiently problematized: It is not clear why pharmacists are in the first line regarding doping? The examples provided in the beginning of the MS did not indicate that the pharmacists were involved in inadvertent doping.**

***Response to reviewer*:** We have added descriptions of the roles of community pharmacists in tackling issues related to doping in sport:

“According to Tsarouhas et al., 15% of athletes obtained drug products from pharmacies [7]. Athletes may also ask pharmacists for advice on the use of medications or supplements for various conditions, including the management of sports injuries [8]. The International Pharmaceutical Federation (FIP) recognises pharmacists’ roles in preventing doping in sports. It is recommended that pharmacists should update themselves on the World Anti-Doping Code and help athletes to identify products containing substances prohibited by the World Anti-Doping Agency (WADA) [9].”

5. **Another key point is the cultural definition of the pharmacists work in Malaysian that is not defined. Indeed, it is expected the potential role of the pharmacists could change between countries: from physician collaboration, patient counselling to commercial benefits… Please explain how pharmacists work in Malaysian: are there able to sell drugs without physicians order? Is there drugs that are free to sell even they are prohibited in the WADA list?**

***Response to reviewer:*** In Malaysia, there are different categories of medications that can be dispensed by community pharmacists i.e.:

- Group A Can only be sold by a licensed wholesaler to a pharmacist or to another licensed wholesaler or by a licensed wholesaler to be immediately exported to a purchaser outside Malaysia.
- Group B Can be dispensed only against prescription of a Registered Medical Practitioner, Dentist, or Veterinary Surgeon, as the case may be and with the prescription in the correct form as required by the law.
- Group C Can only be sold as a dispensed medicine with entry in the Prescription Book.
- Group D Can only be sold as a dispensed medicine with an entry in the Poisons Book.
- Part II Poisons Retail sale restricted to Poison Licence Holder. Labelling requirements only.
- NP- Non-scheduled Poisons Non-scheduled poisons or over the counter products for retail sale.

*Source:* MIMS Malaysia (<https://www.mims.com/malaysia/viewer/html/poisoncls.htm>)

It depends on:

- Whether the drugs have legitimate medical uses.
- The potential of the drugs to be misused by athletes to gain unfair advantages in sports.

*Note:* This information is not included in the manuscript because it is not directly related to the study findings.

*In the original version of the manuscript, we have already highlighted in the discussion the misuse of different classes of drugs that could be prevented by interventions by community pharmacists:*

However, it is worth pointing out that many of the WADA-prohibited drugs are commonly used in medical treatments and are therefore not banned in these settings e.g., insulin is not well known for its performance-enhancing properties, while beta-blockers are prohibited in-competition for only certain sports such as archery and shooting, which may explain why these substances were less recognised by pharmacists as prohibited substances.

6. **Is there a commercial activity linked to the price of drugs that offers potential differential benefits between pharmacists’ office? There is no review of research and most of the references are only proposed in the discussion.**

***Response to reviewer:*** This is an interesting point. However, the commercial activity linked to the price is irrelevant to this study.

7. **In addition, the question of assessment of the knowledge is not considered: what types of knowledge? How they could be assessed?**

***Response to reviewer:*** We have expanded the description of how we adapted the questionnaire from previous studies:

“The knowledge section included questions that were adapted from a previous survey that assessed the readiness of community pharmacists as counsellors for athletes [10]. The questions can be classified into five main domains [1,11,12]: 1) Prohibited substances in sports; 2) The roles of WADA; 3) Doping violations; 4) General knowledge of doping; 5) Doping cases in Malaysia.”

8. **In addition in the introduction, it will be also interesting to indicate their potential role for doping violation rule regarding trafficking of prohibited substances to athletes and unintentional intake of a prohibited substances.**

***Response to reviewer:*** This is an interesting point. However, community pharmacists play no roles in handling anti-doping rule violations. So, this is irrelevant to this study.

In regard to unintentional intake of prohibited substances, we have added the following into our introduction:

“Athletes may become ill or injured, or suffer from chronic medical conditions [13], which may necessitate the use of medications that are banned in sports. For instance, in Japan, many over-the-counter medications contain prohibited substances; this explains the high rate of unintentional doping in the country [14].”

“Inadvertent doping also involves the use of nutritional supplements among athletes. In prior studies, 55% to 98% of athletes were reported to be consuming nutritional supplements [15,16]. Inadvertent doping results when supplement labels contain misinformation that misleads athletes to falsely believe that prohibited products are allowed in sports [17].

9. **To resume, this article did not provide a sufficient report of what you know now and what we need to know that justify this study.**

***Response to reviewer:*** Referring to our first response, the aim of the study is to obtain baseline findings that will inform the design of future studies involving specific interventions among pharmacists.

**Reviewer #2:**

10. **Introduction: Written well.**

***Response to reviewer:*** Noted with thanks.

11. **Methods:
    Data Collection: How authors ensured that participants have not referred internet while giving knowledge-based questions?**

***Response to reviewer:*** In the respondent explanatory statement, we remind the respondents that their honesty in answering the questionnaire is crucial to ensuring the validity of the study findings. We conducted the study based on ‘trust’ and have faith in pharmacists’ professionalism and ethical integrity.

Nevertheless, this is an important point. We have briefly discussed it under ‘Limitations’.

“The first limitation of the study relates to the use of self-administered questionnaires. Although the explanatory statement clearly indicates that the respondents should answer the knowledge-based questions honestly without referring to any resources, some might not have followed the guidelines. This may have led to inaccuracy in the assessment of the respondents’ knowledge.”

12. **Reviewer #2: Discussion: Subheading may not requires under discussion.**

***Response to reviewer:*** All the subheadings were removed.

13. **Reviewer #2: Conclusion: Please remove the limitations which are mentioned under the heading of conclusion. Make separate heading for limitation.**

***Response to reviewer:*** We have created a separate subsection for the study limitations.

14. **Reviewer #2: References: Internet based references please make sure are active and referenced in appropriate manner, if reader would like to visit internet based links. these internet based references should be accessible.**

***Response to reviewer:*** The references have been amended accordingly.

**References cited in this letter:**

1. Auersperger I, Topic MD, Maver P, Pusnik VK, Osredkar J, Lainscak M. Doping awareness, views, and experience: a comparison between general practitioners and pharmacists. Wien Klin Wochenschr. 2012;124(1-2):32-8.

2. Howard MS, DiDonato KL, Janovick DL, Schroeder MN, Powers MF, Azzi AG, Lengel AJ. Perspectives of athletes and pharmacists on pharmacist-provided sports supplement counseling: An exploratory study. Journal of the American Pharmacists Association. 2018 Jul 1;58(4):S30-6.

3. Starzak, D., Derman, W., Mckune, A. & Semple, S. 2016. Anti-Doping Knowledge and Opinions of South African Pharmacists and General Practitioners. Journal of Sports Medicine and Doping Studies 6: 1-7.

4. Teong WW, Ng YK, Paraidathathu T, Chong WW. Job satisfaction and stress levels among community pharmacists in Malaysia. Journal of Pharmacy Practice and Research. 2019 Feb;49(1):9-17.

5. Selvaraj A, Redzuan AM, Hatah E. Community pharmacists’ perceptions, attitudes and barriers towards pharmacist-led minor ailment services in Malaysia. International journal of clinical pharmacy. 2020 Apr;42(2):777-85.

6. Francis J, Toh LS, Sellappans R, Loo JS. Awareness of osteoporosis risk assessment tools and screening recommendations among community pharmacists in Malaysia. International journal of clinical pharmacy. 2021 Jan 28:1-9.

7.Tsarouhas K, Kioukia–Fougia N, Papalexis P, Tsatsakis A, Kouretas D, Bacopoulou F, Tsitsimpikou C. Use of nutritional supplements contaminated with banned doping substances by recreational adolescent athletes in Athens, Greece. Food and chemical toxicology. 2018 May 1;115:447-50.

8. Howard, M. S., Didonato, K. L., Janovick, D. L., Schroeder, M. N., Powers, M. F., Azzi, A. G. & Lengel, A. J. 2018. Perspectives of Athletes and Pharmacists on Pharmacist-Provided Sports Supplement Counseling: An Exploratory Study. J Am Pharm Assoc (2003) 58(4s): S30-S36.e32.

9. Awaisu, A., Mottram, D., Rahhal, A., Alemrayat, B., Ahmed, A., Stuart, M. & Khalifa, S. 2015. Research Knowledge and Perceptions of Pharmacy Students in Qatar on Anti-Doping in Sports and on Sports Pharmacy in Undergraduate Curricula. 79.

10. Chiang LM, Hatah E, Shamsuddin AF. The readiness of community pharmacists as counsellors for athletes in addressing issues of the use and misuse of drugs in sports. Lat. Am. J. Pharm. 2018 Jan 1;37(5):1049-55.

11. The World Anti Doping Code: The 2019 Prohibited List, (2019).

12. WORLD ANTI-DOPING CODE 2015 with 2019 amendments, (2019).

13. Overbye, M. & Wagner, U. 2013. Between Medical Treatment and Performance Enhancement: An Investigation of How Elite Athletes Experience Therapeutic Use Exemptions. 24.

14. Shibata, K., Ichikawa, K. & Kurata, N. 2017. Knowledge of Pharmacy Students About Doping, and the Need for Doping Education: A Questionnaire Survey. BMC Res Notes 10(1): 396.

15. Balaravi, B., Mei Qi, C., Wen Jin, C., Quah Lw, S., Ramadas, A. & Karppaya, H. 2017. Knowledge and Attitude Related to Nutritional Supplements and Risk of Doping among National Elite Athletes in Malaysia. 23.

16. Somerville, S. J., Lewis, M. & Kuipers, H. 2005. Accidental Breaches of the Doping Regulations in Sport: Is There a Need to Improve the Education of Sportspeople? Br J Sports Med 39(8): 512-516; discussion 516.

17. Davar, V. 2012. Nutritional Knowledge and Attitudes Towards Healthy Eating of College-Going Women Hockey Players. 37.

Sincerely,

**Dr Adliah Mhd Ali**

Faculty of Pharmacy,

Universiti Kebangsaan Malaysia,

Jalan Raja Muda Abdul Aziz,

50300 Kuala Lumpur,

Malaysia.

Telephone: +603-92897964

Fax: +603-26983271

Email: [adliah@ukm.edu.my](mailto:adliah@ukm.edu.my)
